# *Nitrosomonas stercoris* sp. nov., a Chemoautotrophic Ammonia-Oxidizing Bacterium Tolerant of High Ammonium Isolated from Composted Cattle Manure

**DOI:** 10.1264/jsme2.ME15072

**Published:** 2015-07-04

**Authors:** Tatsunori Nakagawa, Reiji Takahashi

**Affiliations:** 1 College of Bioresource Sciences, Nihon University, 1866 Kameino, Fujisawa, Kanagawa 252–0880, Japan

**Keywords:** high ammonium-tolerant, cattle manure, ammonia-oxidizing bacteria, compost, *Nitrosomonas stercoris*

## Abstract

Among ammonia-oxidizing bacteria, *Nitrosomonas eutropha*-like microbes are distributed in strongly eutrophic environments such as wastewater treatment plants and animal manure. In the present study, we isolated an ammonia-oxidizing bacterium tolerant of high ammonium levels, designated strain KYUHI-S^T^, from composted cattle manure. Unlike the other known *Nitrosomonas* species, this isolate grew at 1,000 mM ammonium. Phylogenetic analyses based on 16S rRNA and *amoA* genes indicated that the isolate belonged to the genus *Nitrosomonas* and formed a unique cluster with the uncultured ammonia oxidizers found in wastewater systems and animal manure composts, suggesting that these ammonia oxidizers contributed to removing higher concentrations of ammonia in strongly eutrophic environments. Based on the physiological and phylogenetic data presented here, we propose and call for the validation of the provisional taxonomic assignment *Nitrosomonas stercoris*, with strain KYUHI-S as the type strain (type strain KYUHI-S^T^ = NBRC 110753^T^ = ATCC BAA-2718^T^).

Chemoautotrophic ammonia-oxidizing bacteria (AOB) that oxidize ammonium to nitrite are found not only in natural ecosystems such as soils, sediment, freshwater, and marine water, but also in agricultural soils, industrial wastewaters, and manure produced from livestock (25–27). AOB play an important role in the global nitrogen cycle because the first step of the nitrification is mediated by AOB. AOB are comprised of the genera *Nitrosospira* and *Nitrosomonas* in the *Betaproteobacteria* (25, 30) and *Nitrosococcus* in the *Gammaproteobacteria* (1). Other than AOB, ammonia-oxidizing archaea have recently been discovered predominantly in natural environments (11, 17, 20, 23, 28), indicating important microorganisms that are responsible for the global nitrogen cycle. A higher concentration of ammonium generally inhibits the growth of AOB and ammonia-oxidizing archaea possibly because free ammonia (NH_3_) is a toxic compound for most cells (2) or the product nitrite may be inhibitory. However, high ammonium-tolerant AOB have been cultivated from activated sludge (24, 32) and biologically deodorizing systems (29). Although the maximum ammonium tolerance of known AOB is 600 mM from bacteria such as *Nitrosomonas eutropha*, isolated from municipal sewage (15), and *Nitrosomonas* sp. IWT514, isolated from biologically deodorizing plants (29), to the best of our knowledge ammonium-tolerant AOB have not yet been isolated from manure produced by livestock.

Animal manure composts are used for soil amendments. A large amount of organic nitrogenous compounds is mineralized to ammonia during the composting process. Most of the ammonia is vaporized during the high temperature and high pH conditions of the first step of the composting process (8). Some of the residual ammonia is converted to nitrate by microbial nitrification during the second step of the composting process. Molecular analyses based on 16S rRNA and ammonia monooxygenase A subunit (*amoA*) genes suggest that AOB phylogenetically related to *Nitrosomonas europaea* and *Nitrosomonas eutropha* are involved in the conversion of ammonia to nitrite within manure composts (18, 22, 36–38). In the present study, we physiologically and phylogenetically characterized a novel ammonia-oxidizing bacterium belonging to the genus *Nitrosomonas*, which was isolated from the surface layer of a cattle manure pile during the final step of the composting process.

## Materials and Methods

### Cultivation

The surface layer of composted cattle manure was collected from a composting plant at Nihon University in Fujisawa, Kanagawa, Japan, on 7 April 2007. The temperature of the surface layer of the manure pile was 29°C. The following 2-(4-[hydroxyethyl]-1-piperazinyl) ethanesulfonic acid (HEPES)-trace elements medium modified from HEPES medium (9) was used for the cultivation of strain KYUHI-S^T^. The medium was composed of HEPES (11.92 g L^−1^, 50 mM), KH_2_PO_4_ (0.5 g L^−1^), NaHCO_3_ (0.5 g L^−1^), MgSO_4_·7H_2_O (0.1 g L^−1^), (NH_4_)_2_SO_4_ (2.5 g L^−1^, equivalent to 38 mM NH_4_^+^), CaCl_2_·2H_2_O (5 mg L^−1^), EDTA-Fe (III) (1 mg L^−1^), 5 mL of bromothymol blue solution (bromothymol blue 1 mg mL^−1^, Na_2_HPO_4_ 945 mg mL^−1^, and K_2_PO_4_ 0.49 mg mL^−1^ in deionized water), and 5 mL of trace elements (NaMoO_4_·2H_2_O 10 mg, MnCl_2_·4H_2_O 20 mg, CoCl_2_·6H_2_O 0.2 mg, CuSO_4_·5H_2_O 2 mg, ZnSO_4_·7H_2_O 10 mg, FeSO_4_·7H_2_O 770 mg, and EDTA·2Na 1.03 g in 1,000 mL of deionized water). The pH of the HEPES-trace elements medium was adjusted to 8.2±0.2. One gram of the collected manure was added to 10 mL of sterilized HEPES-trace elements medium in a sterilized test tube closed with a silicon cap and then mixed well. Two milliliters of this suspension was transferred to a sterilized test tube containing 8 mL of sterilized HEPES-trace elements medium, mixed well, and then serially diluted (5-fold) to the 5^−10^ dilution level. The test tubes were incubated at 30°C in the dark and shaken by hand for a few seconds per week. The concentration of nitrite in the culture medium was determined colorimetrically with Griess-Ilosvey reagent (10). The incubated medium in which nitrite production was observed at the highest dilution level was serially diluted (5-fold) until the 5^−10^ dilution level with new medium and then incubated. After the end point dilution step was repeated three times, ammonia-oxidizing cultures were inoculated in a 100-mL Erlenmeyer flask containing 30 mL of HEPES-trace elements medium and then incubated at 30°C in the dark without shaking.

In order to obtain an isolate of ammonia-oxidizing bacterium strain KYUHI-S^T^, the standard dilution plating technique at 30°C on HEPES-trace elements medium solidified with 1% (w/v) gellan gum (Phytagel^™^; Sigma Aldrich, St. Louis, MO, USA) (33) was performed. Each colony was picked up with a sterilized fine glass stick, transferred to a test tube containing 5 mL of the HEPES-trace elements medium, and then incubated at 30°C in the dark without shaking. Three milliliters of cultured medium, in which nitrite was detected after the incubation, was inoculated in a 100-mL Erlenmeyer flask containing 30 mL of HEPES-trace elements medium and then incubated at 30°C in the dark without shaking. This dilution plating was repeated one more time. The purity of the final culture was confirmed by a microscopic examination, a PCR-denaturing gradient gel electrophoresis (DGGE) analysis (19) of the 16S rRNA gene using a primer set EUB341F-GC (5′-CGCCCGCCGCGCCCCGCGCCCGTCCCGCCGCCCCCGCCCGCCTACGGGAGGCAGCAG-3′) (19) and 907R (5′-CCGTCAATTCMTTTGAGTTT-3′) (13) for *Bacteria*, and incubations of each heterotrophic agar plate, 2.1% (w/v) YM broth (Difco^™^, BD, Franklin Lakes, NJ, USA), 0.8% (w/v) nutrient broth (Difco^™^, BD), and 3% (w/v) malt extract (Oriental Yeast, Tokyo, Japan), and heterotrophic liquid media containing 0.8% (w/v), 0.08% (w/v), and 0.008% (w/v) of nutrient broth (Difco). The isolated strain was maintained with 10% (v/v), and transferred to fresh 30 mL of HEPES-trace elements medium (38 mM NH_4_^+^, 50 mM HEPES) in a 100-mL Erlenmeyer flask at 30°C, when the concentration of nitrite increased to approximately 8 mM during the incubation.

### Microscopic observations

Cells were observed under a phase-contrast microscope and epi-fluorescent microscope (Axioplan 2; Zeiss, Jena, Germany). Gram staining was carried out using the standard procedure (12).

Pelletized cells of strain KYUHI-S^T^ grown to the late-exponential phase were fixed with 2% (v/v) glutaraldehyde and 1% (w/v) osmium tetroxide, dehydrated by critical point drying with an HCP-2 (HITACHI, Tokyo, Japan), sputter-coated with gold palladium with a JEE-4X (JEOL, Tokyo, Japan), and then observed under a scanning electron microscope (S-3500N; HITACHI) at an accelerating voltage of 30 kV.

Regarding observations with transmission electron microscopy (TEM), the cells of strain KYUHI-S^T^ grown to the late-exponential phase were sandwiched between two copper grids (Nisshin EM, Tokyo, Japan), fixed by rapidly plunging into liquid propane, transferred to 2% (v/v) osmium tetroxide, and embedded in Spurr’s resin for ultrathin sections. Ultrathin sections obtained with a diamond knife on an ultramicrotome were stained with 2% (v/v) uranyl acetate and lead stain solution (Sigma Aldrich). Cell pellets were fixed with 1% (v/v) paraformaldehyde and 1% (v/v) glutaraldehyde in a 0.05 M cacodylate buffer, and negatively stained with 1% (w/v) phosphotungstic acid (pH7.0) for the morphological visualization of whole cells by TEM. TEM micrographs were taken with JEM-1200EX (JEOL) at an accelerating voltage of 80 kV.

### Nucleic acid analyses

The cells of strain KYUHI-S^T^, *Nitrosomonas* sp. K1 (9), *Nitrosomonas* sp. IWT202 (29), and *Nitrosomonas* sp. IWT514 (29) were collected by centrifugation. The nucleic acids were then extracted with Lysing Matrix B (MP Biomedicals, Solon, OH, USA) and ISOPLANT II (Nippon Gene, Toyama, Japan). The nearly complete 16S rRNA gene of strain KYUHI-S^T^ was amplified by PCR using the universal bacterial primers 8f (5′-AGAGTTTGATCCTGGC TCAG-3′) (6) and 1492R (5′-GGTTACCTTGTTACGACTT-3′) (16) with the following protocol: 94°C for 5 min and then 30 cycles at 94°C for 1 min, 55°C for 1 min and 72°C for 1 min, with a final extension at 72°C for 9 min. The PCR product purified from agarose gel was cloned into the vector pGEM-T (Promega, Madison, WI, USA), and the eventual plasmid insert was sequenced by a BigDye v.3.1 sequencing kit (Life Technologies, Carlsbad, CA, USA) on a Model 3130xl DNA sequencer (Life Technologies) with the primers T7, SP6, EUB341F (5′-CCTACGGGAGGCAGCAG-3′) (19), and 907R (13). The *amoA* gene fragments of strain KYUHI-S^T^, *Nitrosomonas* sp. K1, *Nitrosomonas* sp. IWT202, and *Nitrosomonas* sp. IWT514 were amplified by PCR using the primer set of *amoA*-1F (5′-GGGGTTTCTACTGGTGGT-3′) and *amoA*-2R (5′-CCCCTC KGSAAAGCCTTCTTC-3′) (27) with the following protocol: 94°C for 5 min and then 35 cycles at 94°C for 30 s, 57°C for 30 s, and 72°C for 45 s, with a final extension at 72°C for 5 min. After PCR products were electrophoresed in an agarose gel to determine the lengths of the amplified PCR products, amplicons were purified with a QIAquick PCR purification kit (QIAGEN, Valencia, CA, USA) prior to sequencing. The fragments of *amoA* genes were sequenced with the primers *amoA*-1F and *amoA*-2R as described above. Nucleotides were manually aligned using the CLUSTAL W program in MEGA5 (34). Phylogenetic analyses were performed with neighbor-joining using MEGA5.

In order to determine the DNA G+C content of the genome with strain KYUHI-S^T^, genomic DNA was extracted from the cells grown in HEPES-trace elements medium. The DNA G+C content of strain KYUHI-S^T^ was determined by the direct analysis of the deoxyribonucleosides using high-performance liquid chromatography (LC-10; Shimadzu, Kyoto, Japan) (14).

### Growth and nitrite production characteristics

In order to determine the doubling time of strain KYUHI-S^T^, the cells were cultivated in a 100-mL Erlenmeyer flask containing 30 mL of the medium including 38 mM ammonium at 30°C and with a pH of 8.2 without shaking (*n*=3). The cell numbers and concentration of nitrite in the medium were monitored during the incubation. The cell number was determined with polycarbonate membrane filters (Type GTBP 0.2 μm filters; Millipore, Billerica, MA, USA), and VECTASHIELD Mounting Medium with DAPI (1.5 μg mL^−1^) (VECTOR Laboratories, Burlingame, CA, USA) as described previously (21). pH was measured with a pH meter (F-52; HORIBA, Kyoto, Japan). To determine the optimal temperature for growth, strain KYUHI-S^T^ was cultivated in a test tube containing 10 mL of HEPES-trace elements medium including 38 mM ammonium without shaking at 4, 10, 20, 25, 30, 37, or 40°C for 11 d (*n*=3), and the nitrite concentration was monitored. In order to determine the optimal pH for growth, strain KYUHI-S^T^ was cultivated in a test tube containing 10 mL of the medium including 38 mM ammonium without shaking at pH values of 3.0, 4.0, 5.0, 6.0, 7.0, 8.0, 9.0, or 10.0 (*n*=3), and the nitrite concentration was monitored for 13 d. In order to investigate the effects of the NaCl concentration on nitrite production and cell growth, strain KYUHI-S^T^ was cultivated in a 10 mL medium containing 0, 80, 200, 400, 600, 800, and 1,000 mM NaCl in HEPES-trace elements medium including 38 mM ammonium at 30°C and a pH of 8.2 for 11 d without shaking (*n*=3). In order to investigate the effects of the NaNO_2_ concentration on cell growth, strain KYUHI-S^T^ was cultivated in 10 mL of medium containing 0.8, 50, 100, 200, and 400 mM NaNO_2_ in HEPES-trace elements medium including 38 mM ammonium at 30°C and a pH of 8.2 for 13 d without shaking (*n*=3). In order to test the maximum ammonium tolerances of strains KYUHI-S^T^, IWT514, and IWT202, the cells were incubated in a 10 mL medium containing 4, 40, 60, 100, 200, 400, 600, 800, 1,000, or 1,200 mM NH_4_^+^ added as (NH_4_)_2_SO_4_ at 30°C and a pH of 8.2 for 27 d without shaking (*n*=3), and nitrite concentrations were monitored. Cell numbers were determined after a 27-d incubation. The reagents of (NH_4_)_2_SO_4_ were added into empty test tubes, and autoclaved prior to the addition of 10 mL of the sterile medium containing 50 mM HEPES, but without (NH_4_)_2_SO_4_. In order to determine the utilization of urea, the strain was cultivated in 10 mL of medium containing 18.9 mM urea without shaking for 7 d (*n*=3). The 10% (v/v) inoculum containing the strain was transferred to each fresh at approximately 8 mM nitrite during the incubation for the above entire growth experiments.

### Fatty acid analysis

The compositions of the major cellular fatty acids of strain KYUHI-S^T^ were analyzed by the gas chromatography software system MIDI (Microbial IDentification, Newark, DE, USA). Strain KYUHI-S^T^ was grown to the late-exponential growth phase in 10 L of HEPES-trace elements medium at 30°C, and cellular fatty acids were extracted from the cells and converted to methyl esters according to the manufacturer’s recommendations of Sherlock Microbial Identification System Version 6.0 (MIDI).

### Nucleotide sequence accession numbers

The 16S rRNA and partial *amoA* gene sequences were submitted to the DDBJ/EMBL/GenBank and have been assigned the following accession numbers: AB900133 (16S rDNA of strain KYUHI-S^T^), AB900134, AB900135, AB900136, and AB900137 (*amoA* genes of strains KYUHI-S^T^, IWT514, IWT202, and K1).

## Results and Discussion

### Enrichment and isolation

**Fig. 1:**
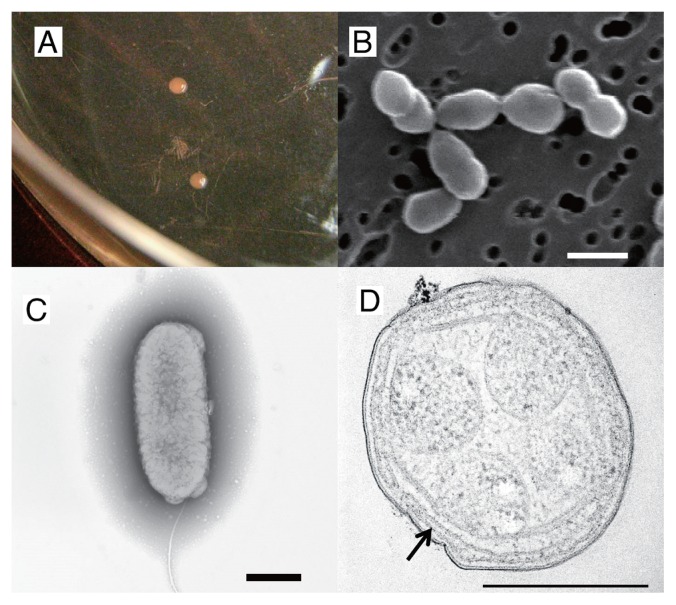
Colonies and cell morphology of *Nitrosomonas stercoris* KYUHI-S^T^. (A) Colonies of strain KYUHI-S^T^ on the gellan gum plate after an 87-d incubation. (B) Scanning electron micrograph of KYUHI-S^T^ cells. Bar=1,000 nm. (C) Transmission electron micrographs of negatively stained strain KYUHI-S^T^. Bar=500 nm. (D) Transmission electron micrographs of ultrathin sections of strain KYUHI-S^T^. Black arrow shows the intracytoplasmic membrane within the periplasm. Bar=500 nm.

Nitrite was detected from the diluted series of primary enrichment cultures until the 5^−7^ dilution level at 30°C after 35 d of cultivation, and the highest diluted medium was transferred to fresh medium. Nitrite was observed from the entire diluted series of the second enrichment cultures until the 5^−10^ dilution level after 35 d of cultivation, and the highest diluted medium was transferred to fresh medium. Nitrite was produced during the entire diluted series of the third enrichment cultures after 35 d of cultivation. The medium at the highest dilution level was transferred to 30 mL of fresh medium in a 100-mL Erlenmeyer flask and then incubated at 30°C. After primary dilution plating on HEPES-trace elements medium solidified with gellan gum, nitrite was detected in 4 out of the 30 colonies picked up. After second dilution plating, nitrite was detected in 5 out of the 30 colonies picked up. The purity of the isolate was verified by microscopic examinations, a DGGE analysis of 16S rRNA gene PCR amplified from the extracted DNA, and inoculation on heterotrophic agar plates. Morphologically, a single cell shape was observed and a sole DGGE band was then confirmed from final cultivation. No growth was detected on agar plates and liquid media for heterotrophs. These results verified that the culture was free of heterotrophs and that the ammonia-oxidizing bacterium strain KYUHI-S^T^ was obtained. Strain KYUHI-S^T^ formed an orange-colored colony (0.66±0.16 mm) on the plate (Fig. 1A). Cells of strain KYUHI-S^T^ were Gram-negative, nonmotile, rod to pear-shaped (Fig. 1B), and possessed a polar monotrichous flagellum (Fig. 1C). A thin section of electron micrographs (Fig. 1D) showed the intracytoplasmic membrane. Previous studies reported that the cells of *Nitrosomonas eutropha* N904 (7) and *Nitrosomonas* sp. IWT514 (29) formed a similar peripherally flattened intracytoplasmic membrane. Although the cells of *Nitrosomonas eutropha* N904 possess carboxysomes (7), no carboxysome was observed in the cells of strain KYUHI-S^T^.

### Physiology and ammonium tolerance

The fatty acid composition of strain KYUHI-S^T^ was C_14:0_ 2-OH (1.16%), C_16:0_ (35.84%), and C_16:1_ (61.21%). As shown in Table 1, the two major fatty acids, C_16:0_ and C_16:1_, of strain KYUHI-S^T^ were similar to those of the other known AOB (4). Although the DNA G+C content of strain KYUHI-S^T^ (45.1 mol%) was similar to that of the phylogenetically closer relative, *Nitrosomonas eutropha* (47.9–48.5 mol%) (15), it was distinct from those of *Nitrosomonas europaea* (50.6–51.4 mol%) (15), *Nitrosomonas halophile* (53.8 mol%) (15), and *Nitrosomonas mobilis* (61.2 mol%) (35).

**Fig. 2:**
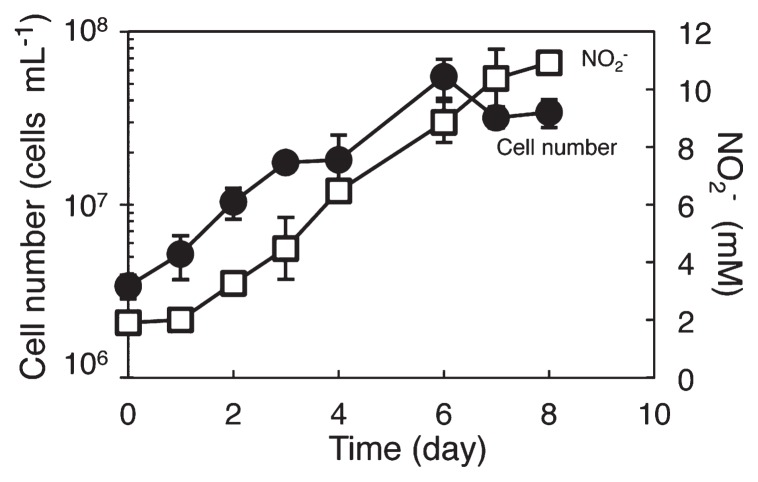
Time-course of changes in the cell number of strain KYUHI-S^T^ and concentration of nitrite in the medium. Bars are standard errors (*n*=3).

**Fig. 3:**
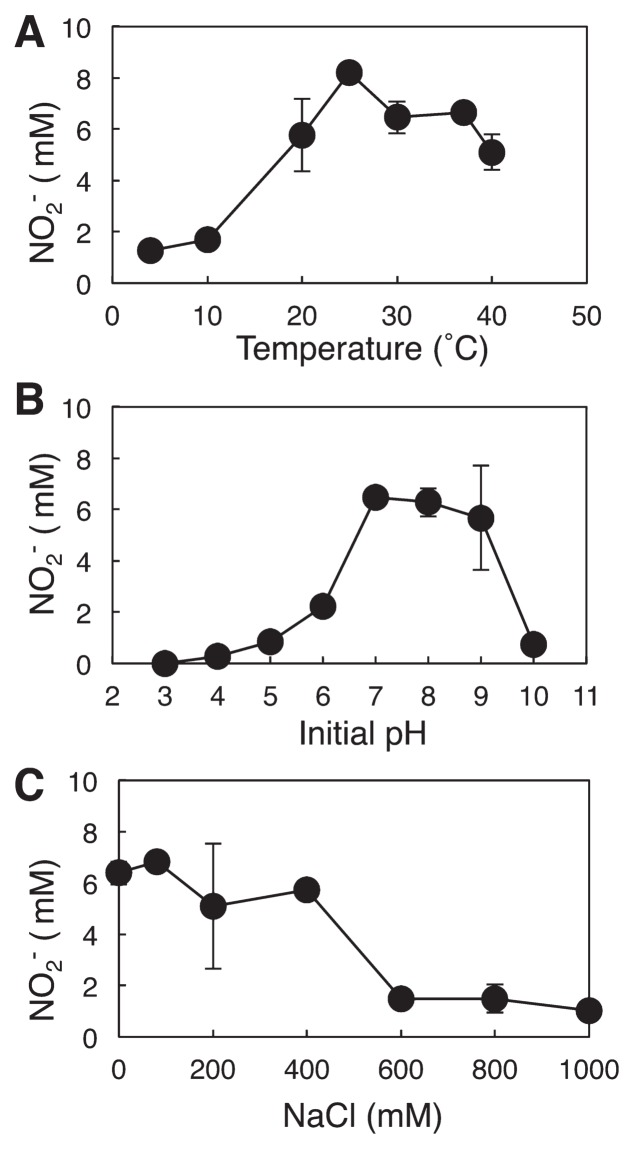
Effects of temperature (A), initial pH (B), and NaCl concentration (C) on nitrite production of strain KYUHI-S^T^. Bars are standard errors (*n*=3).

Strain KYUHI-S^T^ used bicarbonate as the carbon source and oxidized ammonium to nitrite (Fig. 2). The doubling time was approximately one d. Although nitrite production stopped at approximately 21 mM nitrite during the incubation, 17 mM ammonium still remained in the medium. The pH of the medium ranged from 6.5 to 6.2 when the concentration of nitrite was 21 mM. The strain was not sensitive to 200 mM nitrite. Therefore, a decrease in pH may inhibit the activity of strain KYUHI-S^T^. The cell number stained by DAPI decreased at more than 10 mM nitrite in spite of increases in nitrite. The pH of the medium was approximately 7.0 at more than 10 mM nitrite in the incubated culture, and the color of BTB in the medium changed from blue to green. Strain KYUHI-S^T^ grew at temperatures between 20°C and 37°C and with initial pH values between 7.0 and 9.0, with optimum growth at 25°C and an approximate pH of 8.0 (Fig. 3A and B). Strain KYUHI-S^T^ showed no obligate salt requirement (Fig. 3C), similar to other *Nitrosomonas* species retrieved from strongly eutrophic environments (15). However, it showed tolerance to higher NaCl concentrations (maximum 400 mM), similar to *Nitrosomonas eutropha* (15). Strain KYUHI-S^T^ showed no utilization of urea, which was also similar to *Nitrosomonas eutropha* and *Nitrosomonas europaea* (15).

**Fig. 4:**
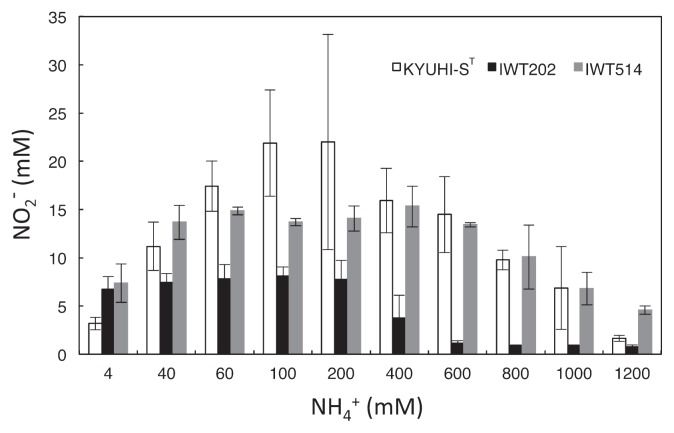
Effects of initial ammonium concentration on nitrite productions of strain KYUHI-S^T^, *Nitrosomonas* sp. IWT514, and *Nitrosomonas* sp. IWT202 after a 27-d incubation. Bars are standard errors (*n*=3).

Strains KYUHI-S^T^ and IWT514 were able to produce nitrite at 1,000 mM and 1,200 mM ammonium, respectively (Fig. 4). However, the maximum tolerance of *Nitrosomonas* sp. IWT202 to ammonium was 400 mM. A previous study demonstrated that *Nitrosomonas eutropha* and *Nitrosomonas eutropha*-like species were tolerant to higher ammonium concentrations, ranging from 600 mM to 800 mM (15). Moreover, the cell numbers of strain KYUHI-S^T^ at 800 mM, 1,000 mM, and 1,200 mM ammonium after a 27-d incubation were 1.3×10^7^±2.5×10^6^ cells mL^−1^, 4.6×10^6^±3.6×10^6^ cells mL^−1^, and 1.1×10^6^±1.8×10^5^ cells mL^−1^, respectively, indicating cell growth occurred at 800 mM and 1,000 mM ammonium. Thus, this result demonstrated that the maximum ammonium tolerances of strains KYUHI-S^T^ and IWT514 were higher than those of the other known AOB. Nitrite production stopped at approximately 20 mM nitrite in the test tube including 200 mM ammonium in spite of 180 mM ammonium not being utilized by cells. Since the strain was not sensitive to 200 mM nitrite, it is possible that the toxicity of nitrite at the lower pH of the medium coupled with ammonia oxidation by the strain may have resulted in reduced nitrite production.

### Phylogenetic analysis

**Fig. 5:**
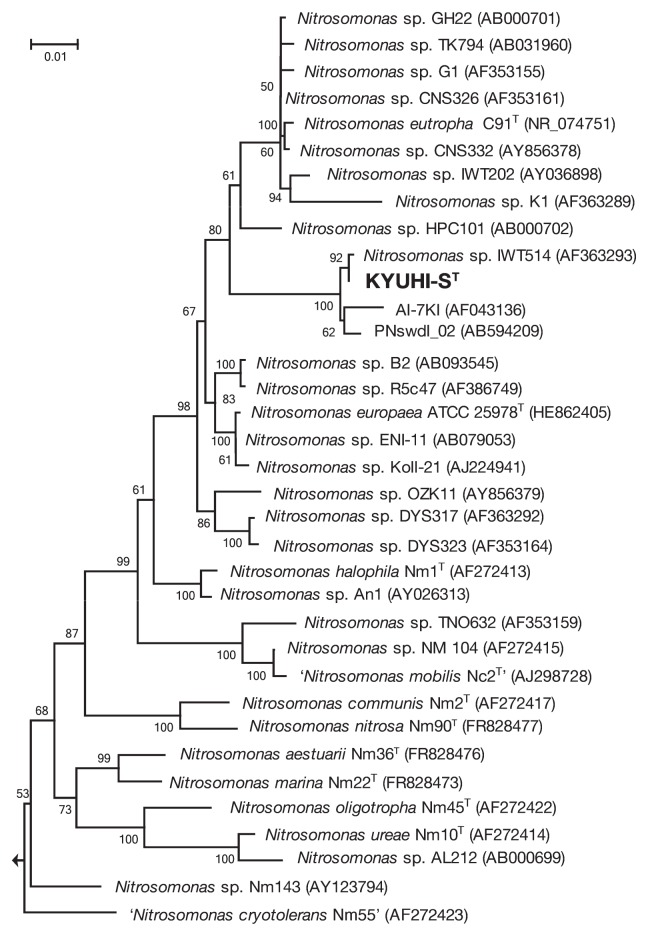
Neighbor-joining phylogenetic trees based on 16S rRNA gene sequences showing the positions of strain KYUHI-S^T^ and representative members of the genus *Nitrosomonas*. Environmental clones AI-7KI (24) and PNswdl_02 (38) were obtained from a wastewater reactor under a high ammonium concentration. Numbers beside branching points indicate bootstrap values determined from 1,000 iterations. Scale bar represents 1 substitution per 100 nucleotides. *Nitrosospira multiformis* ATCC25196^T^ was used as the out-group.

**Fig. 6:**
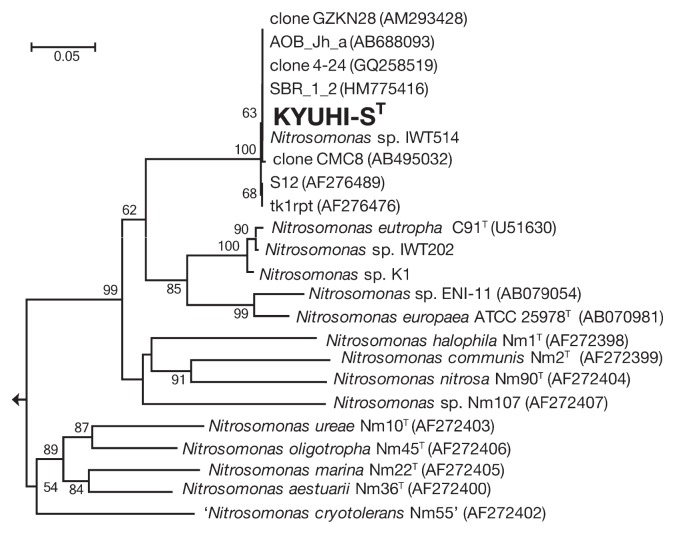
Neighbor-joining phylogenetic trees based on the *amoA* gene sequence showing the positions of strain KYUHI-S^T^ and representative members of the genus *Nitrosomonas*. Environmental clones clone GZKN28 (40), AOB_Jh_a (37), clone4–24 (3), SBR_1_2 (39), clone CMC8 (18), S12 (25), and tk1rpt (25) were obtained from solid waste landfills, animal manure composts, and wastewater treatment plants. Numbers beside branching points indicate bootstrap values determined from 1,000 iterations. Scale bar represents 5 substitution per 100 nucleotides. *Nitrosospira multiformis* ATCC25196^T^ was used as the out-group.

We determined nearly the full-length 16S rRNA gene sequence of strain KYUHI-S^T^. A phylogenetic analysis based on the 16S rRNA gene sequence (Fig. 5) indicated that strain KYUHI-S^T^ should be placed in the genus *Nitrosomonas*. Sequence similarities between strain KYUHI-S^T^ and valid species *Nitrosomonas eutropha* C91^T^ (31), *Nitrosomonas europaea* ATCC25978^T^ (26), *Nitrosomonas halophile* Nm1^T^ (26), and ‘*Nitrosomonas mobilis*’ Nc2^T^ (5) were 96.0%, 95.6%, 94.8%, and 93.4%, respectively. Strain KYUHI-S^T^ was closely related to *Nitrosomonas* sp. IWT514, which was isolated from biologically deodorizing plants (29), with 99.5% similarity, and environmental clones PNswdl_02 (38) and AI-7KI (24) obtained from a wastewater reactor under high ammonium concentrations, with 99.5% and 98.8% similarities, respectively. We also determined the partial *amoA* gene sequences of strain KYUHI-S^T^, *Nitrosomonas* sp. IWT514, *Nitrosomonas* sp. IWT202, and *Nitrosomonas* sp. K1. A phylogenetic analysis based on the *amoA* gene sequence (Fig. 6) indicated that strain KYUHI-S^T^ formed a distinct cluster with *Nitrosomonas* sp. IWT514 with 100% similarity and environmental clones obtained from strongly eutrophic environments such as municipal solid waste landfills: clone GZKN28 (40) with 100% similarity, animal manure: clone AOB_Jh_a (37) with 100% similarity, clone CMC8 (18) with 99.7% similarity, and wastewater treatment plants: clone SBR_1_2 (39) with 100% similarity, clone 4–24 (3) with 100% similarity, clone S12 (25) with 99.7% similarity, clone tk1rpt (25) with 99.7% similarity. Thus, this cluster containing strain KYUHI-S^T^ may be unique to higher ammonia-tolerant AOB.

### Taxonomy

The characterization of strain KYUHI-S^T^ and other *Nitrosomonas* species are summarized in Table 1. The phylogenetic tree based on the 16S rRNA gene sequence (Fig. 5) showed that strain KYUHI-S^T^ was affiliated with the genus *Nitrosomonas* and that *Nitrosomonas eutropha* was a related cluster relative (96% sequence similarity). Similar results were obtained in a phylogenetic analysis based on the *amoA* gene (Fig. 6). A whole-genome study of KYUHI-S^T^ will identify distinctive features between strain KYUHI-S^T^ and *Nitrosomonas eutropha*. In addition, the G+C content of genomic DNA and the sensitivity of the ammonium concentration differentiated strain KYUHI-S^T^ from other *Nitrosomonas* species. Based on these phylogenetic and physiological differences between strain KYUHI-S^T^ and other characterized strains in the genus *Nitrosomonas*, we concluded that strain KYUHI-S^T^ represents a novel species within the genus *Nitrosomonas*, for which the name *Nitrosomonas stercoris* sp. nov. is proposed.

### Description of *Nitrosomonas stercoris* sp. nov

*Nitrosomonas stercoris* (ster’co.ris. L. n. *stercus* manure; L. gen. n. *stercoris* of manure, referring to the source of the isolate). Cells tend to be pleomorphic, rod to pear-shaped (sometimes coccoid). Cells are 0.7–1.2 μm in length and 0.3–0.7 μm in width. Motility not observed. Gram negative. The electron donor used for chemoautotrophic growth is ammonia. No salt requirement and high tolerance for increasing ammonia and salt concentrations. Utilization of urea not observed. The optimum growth conditions are 25°C and initial pH 8.0. The G+C content of genomic DNA of type strain is 45.1 mol%. The type strain, KYUHI-S^T^ (=NBRC 110753^T^ = ATCC BAA-2718^T^), was isolated from composted cattle manure at Nihon University in Fujisawa, Kanagawa, Japan.

## Figures and Tables

**Table 1:** Phenotypic differentiation of strain KYUHI-S^T^ and other species of the genus *Nitrosomonas*

Characteristic	*N. stercoris* KYUHI-S^T^	*N. eutropha* Nm57^T^(C91^T^)	*N. europaea* Nm50^T^
Maximum ammonium tolerance (mM)	1000	600	400
Utilization of urea	—	—	—
NaCl requirement	—	—	—
DNA G+C content (mol%)	45.1	48.2	50.7
Major fatty acid	C_16:0_, C_16:1_ *ω*7*c* / *ω*6c*	C_16:0_, C_16:1_ *ω*7*c*	C_16:0_, C_16:1_ *ω*7*c*
Origin	Matured compost, Fujisawa, Japan	Municipal sewage, Chicago, USA	Soil, USA
References	This study	4, 15	4, 15

—, negative.

* group of two fatty acids that could not be separated with the MIDI system.
